# Influence of tinnitus annoyance on hearing-related quality of life in cochlear implant recipients

**DOI:** 10.1038/s41598-022-18823-3

**Published:** 2022-08-24

**Authors:** Kelly K. S. Assouly, Remo A. G. J. Arts, Petra L. Graham, Bas van Dijk, Chris J. James

**Affiliations:** 1 Department of Otorhinolaryngology and Head & Neck Surgery, University Medical Center Utrecht, Utrecht, The Netherlands; 2 University Medical Center Utrecht Brain Center, Utrecht University, Utrecht, The Netherlands; 3 Cochlear Technology Centre Belgium, Mechelen, Belgium; 4 Cochlear Benelux NV, Mechelen, Belgium; 5 School of Mathematical and Physical Sciences, Macquarie University, North Ryde, NSW Australia; 6 Cochlear France SAS, Toulouse, France

**Keywords:** Quality of life, Auditory system

## Abstract

Tinnitus is a common symptom in cochlear implant (CI) recipients. There is no clear evidence of the influence of tinnitus on hearing-related quality of life (QoL) in this population. The aim of this study was to assess the relationship between hearing-related QoL measured by the Speech, Spatial and Qualities of Hearing scale (SSQ12) and tinnitus annoyance or perceived change in tinnitus annoyance after cochlear implantation. The study sample consisted of 2322 implanted adults across France, Germany, Ireland, Italy, the Netherlands, Sweden and the United Kingdom. Information relating to QoL measured using the SSQ12 and tinnitus annoyance and change in tinnitus annoyance, assessed using single-item questions, were collected one or more years post-implantation. The relationship between SSQ12 score and tinnitus annoyance or change in tinnitus annoyance was analysed using linear models adjusted for age and unilateral versus bilateral implants. Tukey pairwise tests were used to compare mean SSQ12 scores across levels of tinnitus annoyance and changes. Tinnitus prevalence was 33.9% post-implantation. Recipients with tinnitus had a significantly lower SSQ12 score than recipients without tinnitus. SSQ scores varied significantly with tinnitus annoyance, age and unilateral versus bilateral implants. Overall, CI recipients who experienced less bothersome tinnitus reported better hearing-related QoL. Healthcare professionals should be aware of the influence of tinnitus on CI recipients’ hearing to manage patient expectations.

## Introduction

Tinnitus is the perception of sound in the ears or in the head without an external stimulus^1^. It is reported in 10–15% of the general adult population, and the prevalence increases with age^2–4^. Tinnitus does not only vary in terms of sound perception and location but also in terms of distress. Some people are not bothered by tinnitus at all, whereas others experience it as bothersome and debilitating. Up to 3% of the general population experience severe and bothersome tinnitus resulting in a substantial reduction in their quality of life^2,5^.

Hearing impairment is the most common risk factor associated with tinnitus^6,7^. People with severe to profound hearing loss can get hearing benefit from a cochlear implant (CI) when hearing aids no longer provide a sufficient level of speech understanding in everyday situations. Amongst CI candidates, pre-implant tinnitus prevalence ranges from 52 to 86%^8–10^. The cochlear implant primarily aims to partially restore hearing by providing electrical stimulation to the auditory nerve. Tinnitus reduction can be experienced as a beneficial secondary effect of cochlear implantation^8,9,11–13^. While some studies showed that tinnitus loudness, distress or annoyance can be reduced or suppressed after cochlear implantation, others reported that tinnitus can also be worsened in up to 10% of CI recipients, or induced in up to 4% of patients receiving a CI^9,10^. As the prevalence of tinnitus is relatively high in CI candidates and the effect of cochlear implantation on tinnitus distress seems to vary widely between patients, it is of clinical importance to further investigate the impact of tinnitus on CI recipients and how it might influence hearing performance with a CI.

There is no consensus about the influence of tinnitus on hearing performance. Some previous studies showed that tinnitus may interfere with speech perception and reduce hearing performance. Huang et al. compared speech perception between subjects with normal hearing and tinnitus and a control with normal hearing and no tinnitus^14^. The subjects with tinnitus performed significantly worse than subjects without tinnitus. Mertens et al. found that unilateral tinnitus in a single-sided deaf ear reduced speech reception in the non-tinnitus ear^15^. In contrast, Zeng et al. concluded from their data that tinnitus does not interfere with speech perception and perception of external sounds^16^. Therefore, some uncertainties remain about the relationship between tinnitus and hearing performances.

Hearing-related quality of life (QoL) has become a standard outcome measure to quantify the hearing impairment and its associated deficits. Using patient-reported outcome measures (PROMs), several studies found a negative correlation between tinnitus distress and hearing-related QoL in adult CI recipients, meaning that an increase in perceived tinnitus distress correlated with a decrease in hearing-related QoL^10,17–19^. Two studies showed that pre-operative tinnitus was associated with poorer hearing-related QoL outcomes post-implantation^20,21^. In a cohort of 210 adult CI recipients, Opperman et al. showed that both pre- and post-implantation tinnitus were predictive of poorer hearing-related QoL^19^.

Although hearing-related QoL seems related to tinnitus presence and distress, literature does not provide clear evidence for this association. Studies assessing this association used small sizes, which lead large margins of error. Furthermore, previous studies have used different PROMs to assess hearing-related QoL and the impact of tinnitus. Therefore, attempts to merge independent studies into a meta-analysis can hardly be performed to provide an objective appraisal of the evidence. Given this limitation, the association should be validated in a large-scale study. A large-scale study has the advantage of estimating the association with high precision, high statistical power and representativeness of the results.

The availability of hearing-related QoL in a large cohort of CI recipients, as in this report, presents an opportunity to investigate this association. In addition, identifying the relationship between tinnitus and hearing-related QoL might be clinically useful to understand the heterogeneity in hearing outcomes in CI recipients and better manage patient’s expectations. In this cross-sectional study, we aimed to assess the relationship between hearing-related QoL measured by the 12-item Speech, Spatial and Qualities of Hearing scale (SSQ12), the level of tinnitus annoyance measured with a multiple choice question, and the perceived change in the level of tinnitus annoyance produced by cochlear implantation.

## Methods

### Study population

Data from subjects implanted with a Nucleus® CI512, CI522 and CI532 cochlear implants (Cochlear Limited, Macquarie University, NSW, Australia) were extracted from a post-market patient survey. This survey was designed to capture data on self-reported hearing performance and potential side-effects in cochlear implant users. Subjects were adults aged ≥ 18 years old at time of implantation with at least 1 year of experience using the device. Subjects were grouped by whole years of follow-up.

### Data collection

The survey was provided digitally through a web-based survey platform, designed by Cochlear Ltd. Registered users of the Nucleus devices who subscribed to a mailing list were invited by email to participate in the voluntary survey between December 2019 and January 2020. Data were collected across seven European countries: France, Germany, Ireland, Italy, the Netherlands, Sweden and the United Kingdom. All materials were translated using a certified translation process^22,23^, verified for cultural appropriateness by a native speaker and thereafter provided in the local language of the participant. All participants consented to share their data with Cochlear, and to complete the survey. The data were extracted from the web-based survey platform and anonymized.

### Survey

The Speech, Spatial and Qualities of Hearing scale (SSQ) measures hearing-related QoL^24^. For the purposes of the survey, the short form of the SSQ, the SSQ12, was used^25^. It consists of twelve items that cover speech understanding, spatial hearing, and other qualities of sound. Each item is scored using a visual analogue scale ranging from 0 (not at all) to 10 (excellent). The item scores are then averaged to yield an SSQ12 “total” score. Validated versions of the SSQ were used if available in any particular language^26–28^.

Next in the survey, tinnitus presence, defined as ringing in the ear, was assessed using a single-item question. Subjects were first asked to report, at the time of the assessment, if they had tinnitus when the CI was active. If they reported tinnitus, they were asked to complete two single-item questions about how bothersome the tinnitus was perceived and how tinnitus changed since implantation. Possible self-reported tinnitus annoyance levels were not at all bothersome; slightly bothersome; quite a bit bothersome; moderately bothersome or extremely bothersome. For those reporting tinnitus, changes in tinnitus annoyance since implantation were assessed using seven different categories: much less bothersome; a little less bothersome; no change; a little more bothersome; much more bothersome; “I don’t recall the condition before surgery” and “I did not experience this condition before surgery”.

Demographics such as age, gender, time period with implant and unilateral versus bilateral implantation were collected from device registration. There were additional questions in the survey about other potential side effects of cochlear implantation (e.g., dizziness) and magnetic resonance imaging (MRI).

### Ethical considerations

The ethics committee of Medizinische Hochschule Hannover (MHH) confirmed on May 2018 that the Medical Research Involving Human Subjects Act did not apply to the study and therefore an ethical waiver was obtained under the number 7896_MPG_23b_K_2018 and no official ethical approval was required. This study was performed according to the declaration of Helsinki. The subjects provided their informed consent to participate in the survey and to use their data after anonymization, which complies with the General Data Protection Regulation (GDPR).

### Statistical analysis

Data analysis was performed using R version 4.1.2 and R Studio version 1.3.1073 (®R Studio). Any normally distributed data were presented as mean and standard deviation (SD). Where data were not normally distributed, data were reported as median and interquartile range (IQR). The distribution of variables was assessed using normal quantile plots.

Fisher’s exact or chi-squared tests were used to determine whether the tinnitus prevalence, differed between time with implant (1 year, 2 years and 3 years or more), age groups and between unilateral and bilateral implantation. Jonckheere-Terpstra tests (nonparametric one-way analysis of variance [ANOVA] for ordered alternatives) were used to determine whether the SSQ12 scores increased or decreased significantly between the ordinal tinnitus annoyance categories and change categories.

Linear regression models were developed to assess the association between tinnitus characteristics (self-reported tinnitus absence or presence, self-reported tinnitus annoyance, self-reported tinnitus annoyance change) and the SSQ12 scores. The usual regression assumptions were checked using a normal quantile plot of the residuals and plots of the residuals versus fitted values. Models were constructed with and without an interaction for time with implant and adjusted for age and unilateral versus bilateral implants as covariate factors. ANOVA tests were used to compare nested regression models to determine the significance of adding predictors. Relative importance of predictors was assessed using the Lindeman, Merenda and Gold’s (LMG) method available in the *relaimpo* R package^29^. Tukey pairwise tests were used to determine which pairs of tinnitus characteristics differed in SSQ12 scores. A p-value lower than 0.05 was considered statistically significant.

## Results

### Study population characteristics

A total of 7387 CI recipients were invited to participate, of which 2322 consented. Table 1 summarizes the study population characteristics. The mean age of the cohort was 57.9 ± 15.7 years, with range 18–95 years. Fifty percent were female and 69.4% were unilaterally implanted.

**Table 1:** Characteristics of study population.

Characteristics	
**Age**	
Mean (SD)	57.9 (15.7)
Range	18–95
**Gender, n (%)**	
Male	1151 (49.6%)
Female	1161 (50.0%)
Missing	10 (0.4%)
**Implantation, n (%)**	
Unilateral	1612 (69.4%)
Left	484 (20.8%)
Right	498 (21.5%)
Unknown side	630 (27.1%)
Bilateral	695 (30.0%)
Missing	15 (0.6%)
**Time with implant, n (%)**	
1 year	429 (18.5%)
2 years	522 (22.5%)
3 years or more	1371 (59.0%)

### Tinnitus annoyance profile

Table 2 summarizes tinnitus characteristics. The presence of tinnitus was reported by 33.9% (787/2322) of responders and proportions were similar by time with implant (chi-squared test, *p* = 0.434, Table 3). A larger proportion of unilateral experienced tinnitus versus bilateral implants (35.5% vs 30.5%, *p* = 0.023, chi-squared test, Table 2) and the proportion of those with tinnitus differed between age groups (chi-squared test, *p* = 0.034, Table 4). Of those reporting tinnitus postoperatively, 11.1% (87/787) rated their tinnitus as not at all bothersome, 59.7% (470/787) considered it slightly or quite a bit bothersome, and 29.2% (230/787) qualified it as moderate to extremely bothersome (Table 2). Among those reporting tinnitus change postoperatively, 44.0% (346/787) of subjects reported a decrease in bothersome tinnitus since implantation and 18.0% (142/787) indicated that tinnitus had become a little or much more bothersome since implantation. No change in tinnitus annoyance since implantation was reported by 25.9% (204/787, Table 2) and 12.0% could not recall experiencing or having tinnitus before implantation.

**Table 2:** Number (%) of subjects reporting tinnitus characteristics by unilateral versus bilateral implants.

	All (*n* = 2322)	Unilateral (*n* = 1612)	Bilateral (*n* = 695)	Missing (*n* = 15)	p-value
No tinnitus	1535 (66.1%)	1040 (64.5%)	483 (69.5%)	12 (80.0%)	**0.023**
Tinnitus	787 (33.9%)	572 (35.5%)	212 (30.5%)	3 (20.0%)	
**Tinnitus annoyance (*n* = 787)**					0.463
Not at all bothersome	87 (11.1%)	65 (11.4%)	22 (10.4%)	0 (0.0%)	
Slightly bothersome	241 (30.6%)	178 (31.1%)	62 (29.2%)	1 (6.6%)	
Quite a bit bothersome	229 (29.1%)	167 (29.2%)	62 (29.2%)	0 (0.0%)	
Moderately bothersome	167 (21.2%)	123 (21.5%)	43 (20.3%)	1 (6.6%)	
Extremely bothersome	63 (8.0%)	39 (6.8%)	23 (10.8%)	1 (6.6%)	
**Tinnitus annoyance change (*n* = 787)**					0.068
Much less bothersome	153 (19.4%)	110 (19.2%)	42 (19.8%)	1 (33.3%)	
A little less bothersome	193 (24.5%)	144 (25.2%)	48 (22.6%)	1 (33.3%)	
No change	204 (25.9%)	148 (25.9%)	56 (26.4%)	0 (0.0%)	
A little more bothersome	72 (9.2%)	60 (10.5%)	11 (5.2%)	1 (33.3%)	
Much more bothersome	71 (9.0%)	52 (9.1%)	19 (9.0%)	0 (0.0%)	
Do not recall the condition before surgery	33 (4.2%)	19 (3.3%)	14 (6.6%)	0 (0.0%)	
Did not experience this condition before surgery	61 (7.8%)	39 (6.8%)	22 (10.4%)	0 (0.0%)	

p-values are from chi-squared tests comparing unilateral and bilateral (i.e. missing category excluded). Bold indicates statistically significant p < 0.05.

**Table 3:** Number (%) of subjects reporting tinnitus relative to implant experience.

	1 year (*n* = 429)	2 years (*n* = 522)	3 or more years (*n* = 1371)	p-value
No tinnitus	295 (69%)	341 (65%)	899 (66%)	0.434
Tinnitus	134 (31%)	181 (35%)	472 (34%)	

p-value is from a chi-squared test.

**Table 4:** Number (%) of subjects reporting tinnitus by age group.

	18–34 (*n* = 222)	35–44 (*n* = 214)	45–54 (*n* = 415)	55–64 (*n* = 596)	65–74 (*n* = 549)	75–95 (*n* = 324)	Missing (*n* = 2)	p-value
No tinnitus	162 (73.0%)	143 (66.8%)	273 (65.7%)	366 (61.4%)	367 (66.8%)	224 (69.1%)	0 (0%)	**0.034**
Tinnitus	60 (27.0%)	71 (33.2%)	142 (34.2%)	230 (38.6%)	182 (33.2%)	100 (30.9%)	2 (100%)	

p-value is from a chi-squared test excluding the missing category. Bold indicates statistically significant p < 0.05.

**Figure 1:**
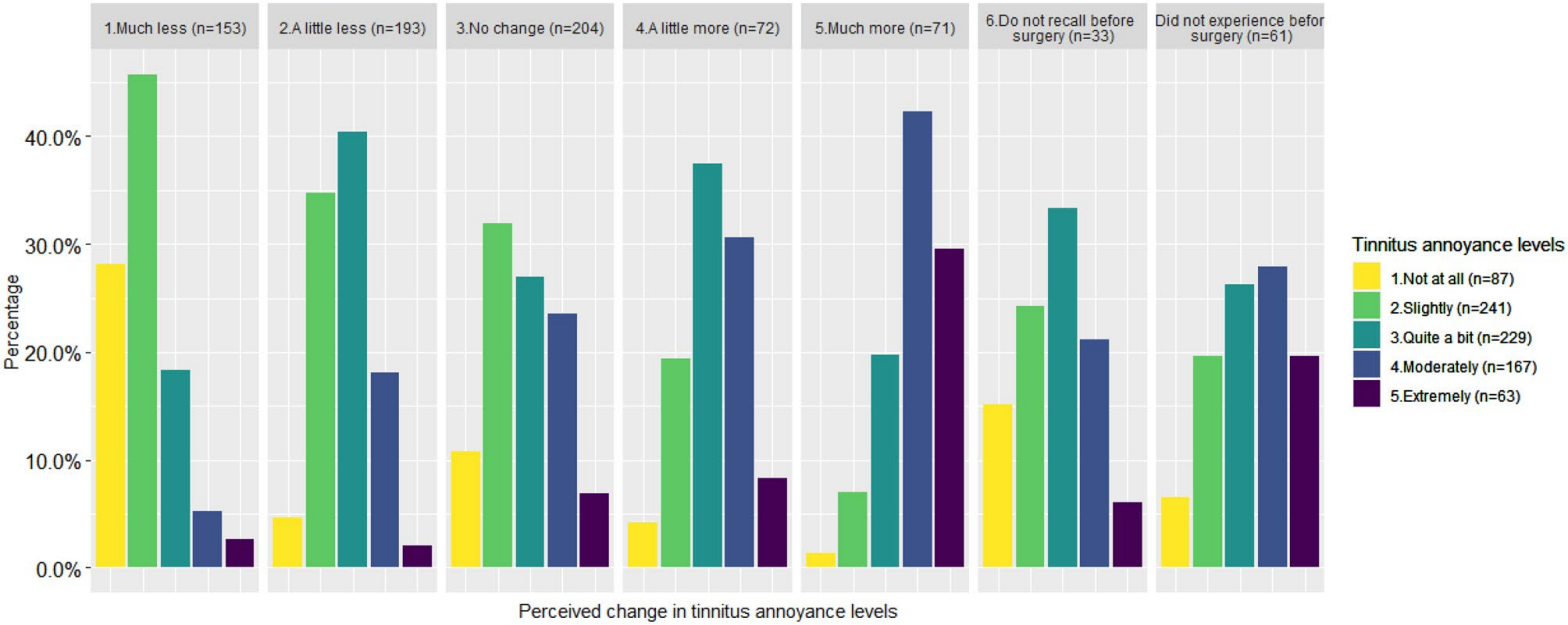
Percentage of recipients in each tinnitus annoyance level within perceived change in tinnitus annoyance groups. The proportion of recipients in each self-reported tinnitus annoyance level differed between perceived change groups (Fisher’s exact test, *p* < 0.001).

Figure 1 shows the percentage of each tinnitus annoyance level within perceived change in tinnitus annoyance. A higher proportion of participants with much more post-implantation tinnitus also have more bothersome tinnitus compared to those with less post-implantation tinnitus (Fisher’s exact test, *p* < 0.001).

### Age and unilateral versus bilateral implants as covariates

There was no significant association between time with implant and SSQ12 scores (ANOVA test, F = 0.705, *p* = 0.49). Older age and unilateral (versus bilateral implants) were significantly associated with lower SSQ12 scores in all models (p < 0.001, Table 5).

**Table 5:** Multiple regression models of the SSQ12 scores.

Coefficient	Parameter estimate	Standard error	t value	p-value	R^2^
**Model 0: age and unilateral versus bilateral implants**					3.4%
Intercept	5.60	0.18	31.14	**< 0.001**	
Age (years)	− 0.02	0.01	− 5.40	**< 0.001**	
Bilateral CI (versus unilateral CI)	0.56	0.10	5.77	**< 0.001**	
**Model 1: tinnitus presence**					5.9%
Intercept	5.85	0.18	32.41	**< 0.001**	
Age (years)	− 0.02	0.01	− 5.44	**< 0.001**	
Bilateral CI (versus unilateral CI)	0.53	0.10	5.48	**< 0.001**	
Tinnitus presence (versus tinnitus absence)	− 0.71	0.09	− 7.79	**< 0.001**	
**Model 2: tinnitus annoyance**					8.4%
Intercept	5.84	0.18	32.78	**< 0.001**	
Age (years)	− 0.02	0.01	− 5.51	**< 0.001**	
Bilateral CI (versus unilateral CI)	0.55	0.10	5.77	**< 0.001**	
Tinnitus level (versus tinnitus absence)					
Not at all bothersome	0.32	0.22	1.44	0.149	
Slightly bothersome	− 0.35	0.14	− 2.45	**0.015**	
Quite a bit bothersome	− 0.90	0.14	− 6.23	**< 0.001**	
Moderately bothersome	− 1.00	0.17	− 5.99	**< 0.001**	
Extremely bothersome	− 2.04	0.26	− 7.71	**< 0.001**	
**Model 3: tinnitus annoyance change**					7.8%
Intercept	5.85	0.18	32.66	**< 0.001**	
Age (years)	− 0.02	0.01	− 5.51	**< 0.001**	
Bilateral CI (versus unilateral CI)	0.53	0.10	5.58	**< 0.001**	
Tinnitus change (versus tinnitus absence)					
Much less bothersome	0.07	0.17	0.39	0.700	
A little less bothersome	− 0.64	0.16	− 4.10	**< 0.001**	
No change	− 0.62	0.15	− 4.02	**< 0.001**	
A little more bothersome	− 1.08	0.25	− 4.35	**< 0.001**	
Much more bothersome	− 1.55	0.25	− 6.24	**< 0.001**	
Do not recall it before surgery	− 1.45	0.36	− 4.02	**< 0.001**	
Did not experience it before surgery	− 1.32	0.27	− 4.92	**< 0.001**	

Bold indicates statistically significant p < 0.05.

### Association between hearing-related QoL and tinnitus status

The association between SSQ12 scores and tinnitus presence is presented in Table 5 (Model 1). Recipients with tinnitus had significantly lower SSQ12 scores than recipients without tinnitus (mean difference − 0.71 [SD: 0.09], F-test, *p* < 0.001, Supplementary Fig. S1) after adjusting for age and presence of unilateral/bilateral implants. Using tinnitus presence as a predictive factor, Model 1 explained significantly more of the variability in SSQ12 scores compared with the simplest Model 0, using only age and unilateral versus bilateral implants as predictors (*Model 0* (R^2^ = 3.4%) *vs Model 1* (R^2^ = 5.9%), ANOVA test, *p* < 0.001, Table 5).

### Association between hearing-related QoL and self-reported tinnitus annoyance

**Figure 2:**
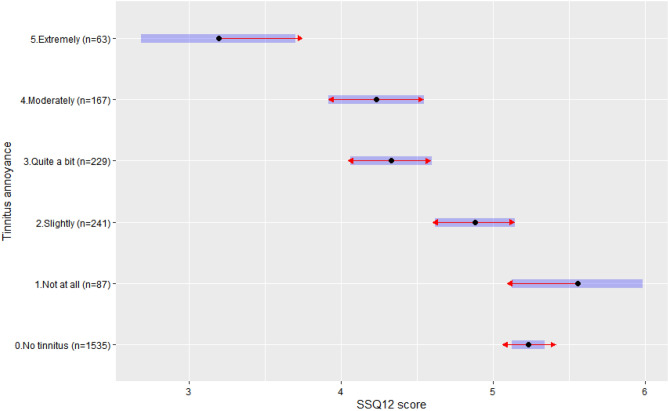
SSQ12 scores by tinnitus annoyance levels. Blue bars are the 95% confidence intervals for the estimated marginal means, the red arrows are for the comparisons among them. If an arrow from one group overlaps with an arrow from another group, the difference is not statistically significant (Tukey tests, p > 0.05).

The association between SSQ12 scores and tinnitus annoyance is presented in Table 5 (Model 2). SSQ12 scores significantly decreased with increased tinnitus annoyance (Jonckheere-Terpstra test, *p* < 0.001, Supplementary Fig. S2). Figure 2 shows Tukey pairwise comparisons of mean SSQ12 scores between annoyance level groups. The mean difference in SSQ12 scores between recipients without tinnitus and recipients rating their tinnitus as *not at all bothersome* was not significant at only -0.32 (SD: 0.22) (Tukey test, *p* = 0.67, Supplementary Table S1). Recipients rating their tinnitus as *not at all bothersome* had significantly higher mean SSQ12 scores than recipients with higher tinnitus annoyance levels (*quite a bit bothersome*: mean difference 1.23 [SD: 0.26], Tukey test, *p* < 0.001; *moderately bothersome*: mean difference 1.32 [SD: 0.27], Tukey test, *p* < 0.001; *extremely bothersome*: mean difference 2.36 [SD: 0.34], Tukey test, *p* < 0.001), except for tinnitus rated as *slightly bothersome* (Supplementary Table S1). Mean SSQ12 scores were significantly lower for tinnitus rated as *extremely bothersome* than for all other tinnitus annoyance levels (Tukey test, *p* < 0.001, Supplementary Table S1). Model 2, explained significantly more of the variability in SSQ12 scores compared to Model 0 (*Model 0 R*^*2*^ = *3.4% vs Model 2 R*^*2*^ = *8.4%,* ANOVA test, p < 0.001).

### Association between hearing-related QoL and perceived changes in tinnitus annoyance

**Figure 3:**
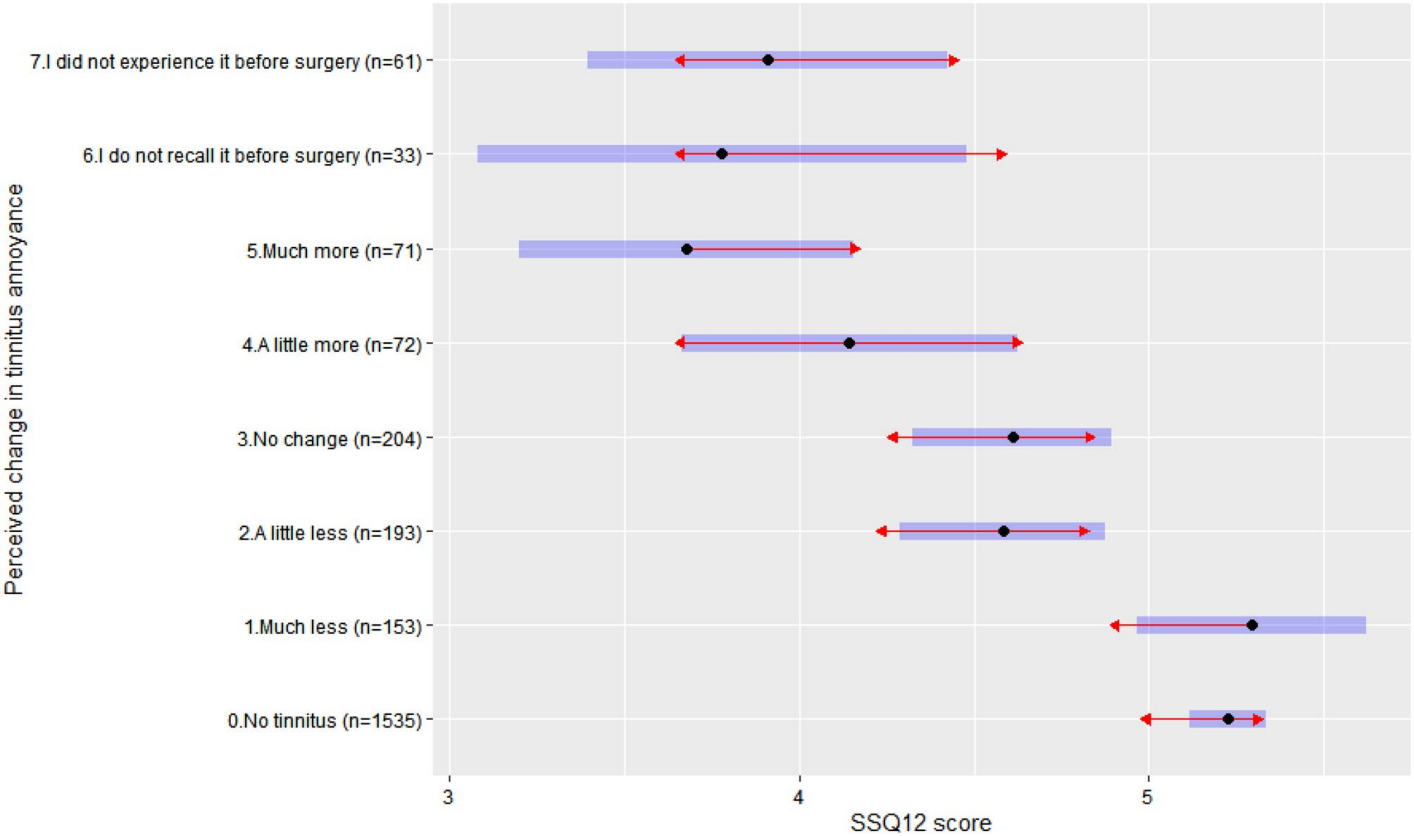
SSQ12 scores by perceived changes in tinnitus annoyance. Blue bars are the 95% confidence intervals for the estimated marginal means, the red arrows are for the comparisons among them. If an arrow from one group overlaps with an arrow from another group, the difference is not statistically significant (Tukey tests, p > 0.05).

The association between SSQ12 scores and perceived changes in tinnitus annoyance since implantation is presented in Table 5 (Model 3) with Tukey pairwise comparisons shown in Fig. 3. SSQ12 scores significantly decreased with increasing perceived changes in tinnitus annoyance (Jonckheere-Terpstra test, *p* < 0.001, Supplementary Fig. S3). Recipients rating their tinnitus as *much more bothersome* had significantly lower mean SSQ12 scores compared with those rating their tinnitus as *much less bothersome* (mean difference − 1.62 [SD: 0.29], Tukey test, *p* < 0.001, Supplementary Table S1). Recipients reporting *no change* in tinnitus annoyance had significantly higher mean SSQ12 scores than the ones with *much more bothersome* tinnitus (mean difference 0.93 [SD: 0.28], Tukey test, *p* = 0.02, Supplementary Table S1) and significantly lower mean SSQ12 scores than those reporting *much less bothersome* tinnitus (mean difference − 0.68 [SD: 0.22], Tukey test, *p* = 0.03, Supplementary Table S1). Recipients with post-operative tinnitus who answered “*I did not experience it before surgery*” had significantly lower mean SSQ12 scores than recipients without post-operative tinnitus (mean difference − 1.45 [SD: 0.36], Tukey test, *p* = 0.001, Supplementary Table S1) and recipients with *much less bothersome* tinnitus (mean difference − 1.38 [SD: 0.32], Tukey test, *p* < 0.001, Supplementary Table S1). Model 3, using perceived change in tinnitus annoyance as a predictor, explained significantly more of the variance in SSQ12 scores compared with Model 0 (*Model 0 R*^*2*^ = *3.4% vs Model 3* R^2^ = 7.8%, ANOVA test, p < 0.001).

### Relative importance of predictors

**Figure 4:**
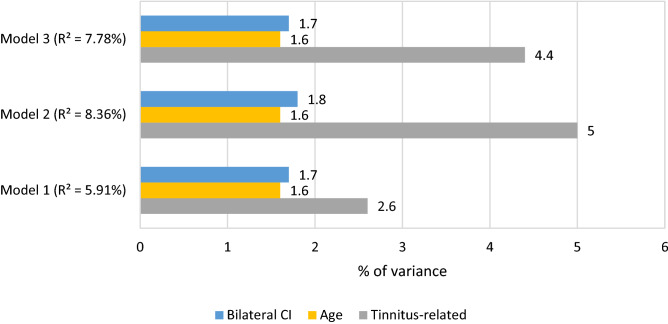
Relative importance of predictors (% of variance) in Model 1, Model 2, Model 3. The method used for relative importance is the Lindeman, Merenda and Gold’s (LMG) method. R^2^ corresponds to the determination coefficient of each model.

Figure 4 summarizes the relative importance of predictive factors in each model. In the three linear models, the predictor related to tinnitus was always most important: tinnitus presence (2.6% in Model 1, LMG method), tinnitus annoyance level (5.0% in Model 2, LMG method) or change in tinnitus annoyance (4.4% in Model 3, LMG method). Age contributed the least to each model (1.6% in Model 1, 2 and 3, LMG method).

## Discussion

Our data-driven approach assessed the relationship between the hearing-related QoL, the level of tinnitus annoyance, and also the perceived change in the level of tinnitus annoyance since cochlear implantation in 2322 adult CI recipients. The analysis demonstrated a statistically significant association between hearing-related QoL assessed by the SSQ12 and both tinnitus annoyance and perceived change in tinnitus annoyance at least one year after implantation. Overall, results suggested that CI recipients who experienced bothersome tinnitus showed worse subjective hearing-related QoL; whereas, CI recipients reporting less bothersome tinnitus since implantation had better hearing-related QoL. The SSQ12 scores were also statistically significantly associated with the age of a recipient and presence of unilateral versus bilateral implants (Table 5). Coefficients of determination (R^2^) showed that only 8.4% of the variance in SSQ12 scores was explained by age, unilateral versus bilateral implantation and tinnitus annoyance levels combined. Therefore, it is highly likely that other important factors might contribute to the variations in SSQ12 scores.

Tinnitus prevalence was 33.9% in our cohort population, which is lower than the expected 40–54% prevalence found in previous studies on adult CI users^10,19,30^. The variability in tinnitus prevalence in CI users can be explained by a non-response bias in previous studies, variations in tinnitus definition and heterogeneous assessment methods to identify the presence of tinnitus^2,31^. For instance, in our study, the presence of tinnitus was estimated on the basis of a self-report when the CI was active, which may differ from the situation when the CI is off. Indeed, tinnitus presence can vary among CI recipients depending on the CI active status (CI on or CI off)^32–34^.

This prevalence significantly varies with age (Table 4), as reported by other authors^2,35–37^. Based on the age group classification used in this study, there was a trend for tinnitus prevalence to increase with age up to 65 years, with 38.6% for the recipients aged between 54 and 65 years, and thereafter reduce for older subject. The same trend was reported by previous studies using different cut offs and age groupings^2,35^. This suggests that tinnitus might not be related to an aging process^38^. However, given the slight differences in proportion of age groups, it is difficult to conclude whether this trend is a true pattern of tinnitus prevalence.

Unilateral CI recipients reported significantly more tinnitus than bilateral CI recipients (Table 2). However, differences in proportion between the two groups is relatively small and questions the relevance of this finding. Moreover, the distribution of age groups varied significantly between unilateral and bilateral CI recipients (chi-squared test, *p* < 0.001, Supplementary Table S2). Therefore, age could play a role as a confounding factor. Levels of tinnitus annoyance and change in tinnitus annoyance do not significantly differ between unilateral and bilateral implants. This might suggest that two implants do not bring significant benefit in terms of tinnitus annoyance compared to a single implant. These findings, however, remain hard to interpret without detailed information about the tinnitus percept (e.g. location), pre-implantation tinnitus outcomes and complementary information on recipients’ hearing loss profiles.

In our study, we used a validated multi-item questionnaire, the SSQ12, to measure hearing-related QoL^25^. The SSQ12 has no question related to tinnitus. The total score ranges from 0 to 10, with 10 indicating a perfect hearing-related QoL. We found that SSQ12 scores significantly decreased with increasing tinnitus annoyance. A mean difference of 2.36 points in SSQ12 scores was found between CI recipients reporting their tinnitus as not at all bothersome and recipients with extremely bothersome tinnitus, which is more than double the clinically significant change of 1.0 SSQ points suggested by the SSQ developers^24^. A mean difference in SSQ12 scores of the same range (2.55 SSQ12 points) was reported in a study investigating the difference between normal-hearing and hearing-loss groups^39^. Likewise, Wyss et al. showed a statistically significant improvement of 2.2 SSQ12 points at one-year post-implant in 1013 auditory implant recipients^40^. Hence, the difference observed between the two extreme levels in tinnitus annoyance post-implantation showed the same magnitude of difference reported between pre-implantation and one-year post-implantation. The high mean differences found between different levels of tinnitus annoyance raises questions about the importance and the impact of tinnitus on hearing-related QoL in CI recipients, which may need further focus in clinics and exploration in future studies.

This study suggested a negative association between tinnitus and hearing-related QoL. When controlling for age and unilateral versus bilateral implants, mean SSQ12 scores were significantly lower in adult CI recipients with tinnitus than in CI recipients without tinnitus. Furthermore, tinnitus annoyance was also negatively associated with hearing-related QoL. The demonstrated association corresponds with the findings of previous studies investigating perceived tinnitus distress in CI recipients^10,17–19^. In a study from Opperman et al., an increase in perceived tinnitus distress was correlated with a decrease in hearing-related QoL based on the Abbreviated Profile of Hearing Aid Benefit (APHAB) scores^19^. This is in line with our findings on perceived changes in tinnitus annoyance where CI recipients who experienced less bothersome tinnitus showed better subjective hearing-related QoL. This might be related to the impact of tinnitus on psychological distress such as stress, coping strategies and depressive and anxiety symptoms^41,42^, but also its impact on speech perception^43^; that is, both psychological and perceptual factors can affect hearing-related QoL. Moreover, SSQ12 scores were not significantly different between adult CI recipients experiencing tinnitus as *not at all bothersome* and CI recipients without tinnitus, highlighting the importance of the degree of tinnitus-related distress over the presence of tinnitus. Further research is needed to fully understand the factors involved in this relation and its implications on CI outcomes.

SSQ12 scores significantly decreased with age (Table 5). The age effect on SSQ scores (including short forms) was already observed in other studies examining minimally hearing-impaired subjects^44^ or CI recipients^10^. Also, SSQ12 scores were significantly higher for bilateral CI recipients compared to unilateral recipients (Table 5). This association should be further investigated to assess if it could be related to other factors such as the implant side^10,20^ or patients’ hearing loss characteristics^45^.

Based on the linear models, 8.4% of the variance in SSQ12 scores was explained by the combination of age, unilateral versus bilateral implants and the level of tinnitus annoyance, with the latter being the most important predictor. Tinnitus annoyance and other tinnitus related characteristics deserve further research to understand what the causal relationship of the association is. The other 92% of the variance in SSQ12 scores could be potentially explained by differences in hearing impairment^45,46^, in speech perception performance^47^, in implant characteristics such as implant side^20^ and in cognitive and linguistic factors^10^. The influence of non-auditory aspects, such as education level^45^, socioeconomic level or additional comorbidities, should also be considered in explaining the variance in SSQ12 scores. Investigating the above characteristics and then adding the tinnitus variable would be a valuable approach to confirm or temper our findings.

The study cohort is derived from a multi-country database collected in a web-based survey platform. This unique database gathers tinnitus and individual characteristics from a large sample of 2322 Nucleus cochlear implant users. Therefore, the findings of the study are expected to be generalizable to the European adult cochlear implant population.

Some methodological issues in this study are worth considering. The first limitation is that the observational study design was not primarily aimed to study the effects of tinnitus. Due to limitations in the number of questions and length of the survey, only three questions were used to assess tinnitus characteristics. From the three tinnitus-related questions used, only one sub-domain of the impact of tinnitus was measured, tinnitus annoyance. Indeed, the impact of tinnitus is complex, often associated with comorbidities such as concentration, sleep or mental health problems and impaired quality of life^48^. Since many different domains can be affected by tinnitus, tinnitus validated questionnaires are often multi-item questionnaires containing sub-scales to assess the different domains of the overall impact of tinnitus^49–52^. In this study, only tinnitus annoyance was assessed, and results cannot be generalized to the overall impact of tinnitus nor the effect of cochlear implantation on tinnitus. Further research is needed to investigate the influence of the overall impact of tinnitus on hearing-related QoL using validated multi-item tinnitus questionnaires. Furthermore, the question related to change in tinnitus annoyance addressed past experience, which could present a potential recall bias. In fact, CI recipients were asked to report the perceived change in tinnitus annoyance since implantation, which corresponded to a time interval of 3 years or more for 472 recipients, potentially increasing recall bias even further. Collecting prospective tinnitus outcomes pre-implantation and post-implantation would provide better insights in order to assess the change in tinnitus annoyance since implantation. In fact, the lack of longitudinal data limits the scope of our study to post-implantation tinnitus experience and prevents us from definitively estimating the effect of cochlear implant on tinnitus annoyance between pre- and post-implantation. Nevertheless, the retrospective design ensures that no adaptation process has taken place between the pre- and post-implantation periods by assessing changes at a given point in time and, thus, controlling for response shift^53^. Finally, we did not fully define tinnitus and other terms in the questions and answers^31^. For instance, the options related to the perceived change in tinnitus annoyance *”I did not experience it before surgery”* and *“I don’t recall it before surgery”* could both be interpreted as reporting tinnitus newly after implantation. Therefore, these deliberations should be taken with caution since we did not have access to the pre-implantation tinnitus report to validate this interpretation.

Considering the clear association between hearing-related QoL and level of tinnitus annoyance, the identification of accompanying tinnitus should be a requirement in the standard CI candidacy evaluation. Clinicians and CI manufacturers should address tinnitus as an important factor to better manage patients’ expectation. This study highlights a need for individualized tinnitus management therapies to be made available within CI counselling and rehabilitation. Further research is needed to determine the underlying mechanisms and relationships. Another aspect that will require further investigation is whether tinnitus annoyance has a direct impact on CI performance such as speech recognition.

## Conclusion

Tinnitus prevalence was 33.9% post-implantation. This prevalence varied with age, with the highest prevalence in middle age. CI recipients with tinnitus had a significantly lower SSQ score than recipients without tinnitus. SSQ scores decreased significantly with increasing level of tinnitus annoyance and age. Overall, CI recipients who experienced less bothersome tinnitus showed better subjective hearing-related QoL. The association of better subjective hearing performance with a positive change in tinnitus annoyance after cochlear implantation should be further explored using a prospective study design and complementary associated factors. Furthermore, healthcare professionals may be well advised to give tinnitus management a higher priority for CI recipients in order for them to maximize their hearing experience.

## Supplementary Information

Supplementary Information.

## Data Availability

The data that support the findings of this study will be available from the corresponding author, KKSA, upon reasonable request.
