# Supplementary material for: Influence of tinnitus annoyance on hearing-related quality of life in cochlear implant recipients
Source: Sci Rep. 2022 Aug 24;12:14423. doi: 10.1038/s41598-022-18823-3 (PMC9402917; doi:10.1038/s41598-022-18823-3)
Supplement: Supplementary file 1 — Supplementary Information. [file 41598_2022_18823_MOESM1_ESM.docx]

**Influence of tinnitus annoyance on hearing-related quality of life in cochlear implant recipients**

Kelly K.S. Assouly^1, 2, 3, *^, Remo A.G.J. Arts^4^, Petra L. Graham^5^, Bas van Dijk^3^, Chris J. James^6^

^1^ Department of Otorhinolaryngology and Head & Neck Surgery, University Medical Center Utrecht, the Netherlands

^2^ University Medical Center Utrecht Brain Center, Utrecht University, Utrecht, The Netherlands

^3^ Cochlear Technology Centre Belgium, Mechelen, Belgium

^4^ Cochlear Benelux NV, Mechelen, Belgium

^5^ School of Mathematical and Physical Sciences, Macquarie University, North Ryde, NSW, Australia

^6^ Cochlear France SAS, Toulouse, France

# **Supplementary Material**


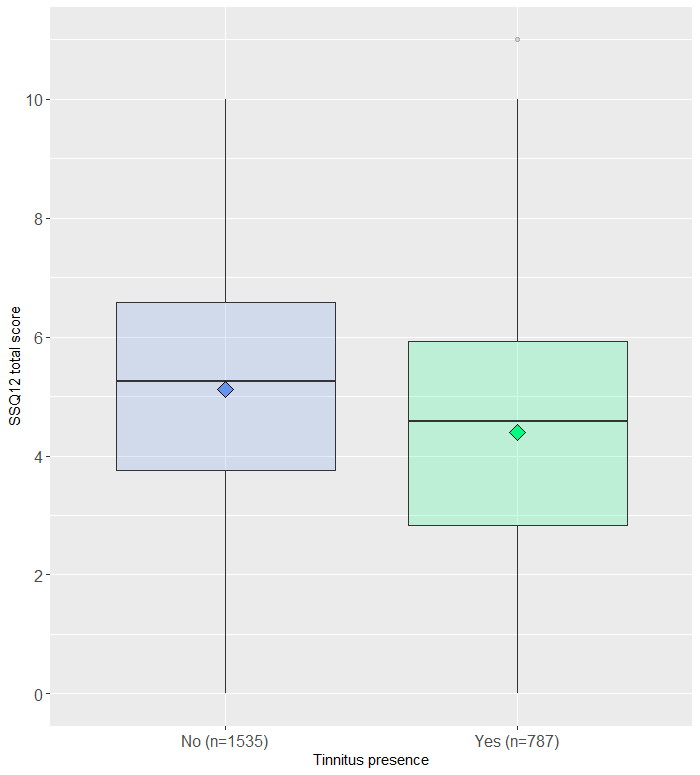


**Supplementary Figure S1**. SSQ12 scores by tinnitus status.


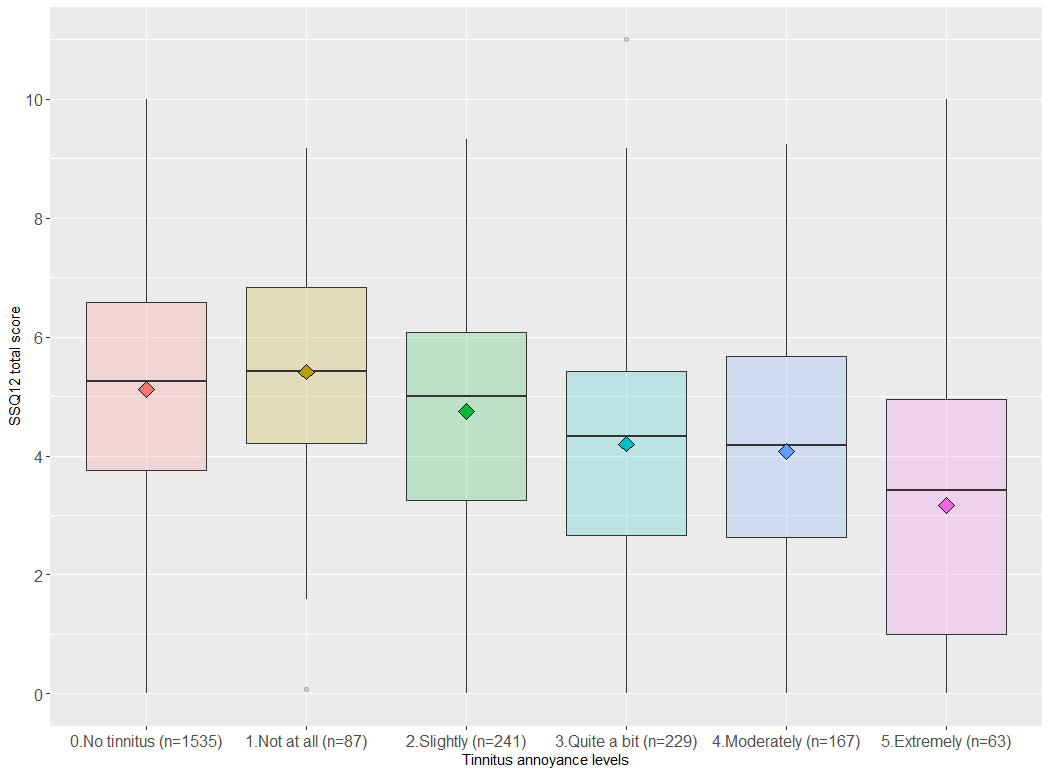


**Supplementary Figure S2**. SSQ12 scores by tinnitus annoyance levels.


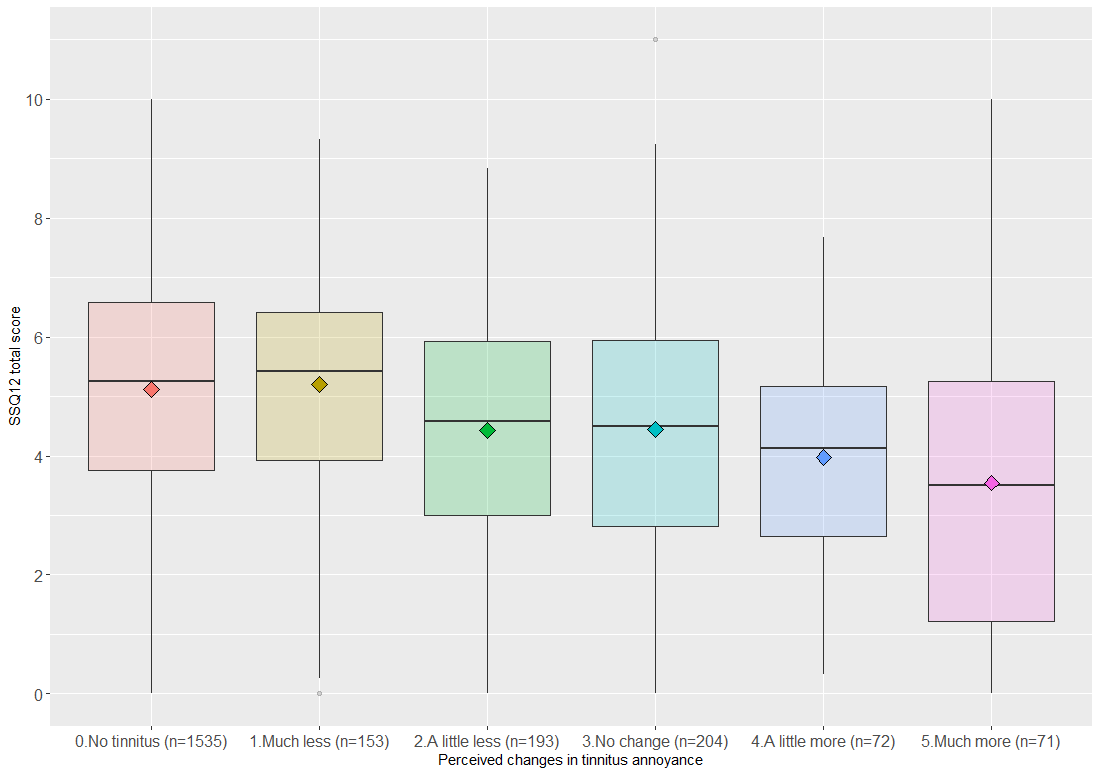


**Supplementary Figure S3**. SSQ12 scores by perceived changes in tinnitus annoyance.

**Supplementary Table S1**. Pairwise comparison tests of SSQ total scores between tinnitus annoyance levels and perceived changes in tinnitus annoyance.

| **Pairwise comparison** | **Difference estimate** | **Standard error** | **t value** | **p-value** |
| --- | --- | --- | --- | --- |
| *Tinnitus annoyance levels* |  |  |  |  |
| No tinnitus – Not at all bothersome | -0.32 | 0.22 | -1.45 | 0.673 |
| No tinnitus – Slightly bothersome | 0.35 | 0.14 | 2.45 | 0.127 |
| No tinnitus – Quite a bit bothersome | 0.90 | 0.14 | 6.23 | **<0.001** |
| No tinnitus – Moderately bothersome | 0.99 | 0.17 | 5.99 | **<0.001** |
| No tinnitus – Extremely bothersome | 2.04 | 0.26 | 7.71 | **<0.001** |
| Not at all bothersome – Slightly bothersome | 0.67 | 0.26 | 2.63 | 0.080 |
| Not at all bothersome – Quite a bit bothersome | 1.23 | 0.26 | 4.77 | **<0.001** |
| Not at all bothersome – Moderately bothersome | 1.32 | 0.27 | 4.90 | **<0.001** |
| Not at all bothersome – Extremely bothersome | 2.36 | 0.34 | 6.97 | **<0.001** |
| Slightly bothersome – Quite a bit bothersome | 0.55 | 0.19 | 2.93 | **0.035** |
| Slightly bothersome – Moderately bothersome | 0.65 | 0.21 | 3.15 | **0.018** |
| Slightly bothersome – Extremely bothersome | 1.69 | 0.29 | 5.81 | **<0.001** |
| Quite a bit bothersome – Moderately bothersome | 0.10 | 0.21 | 0.47 | 1.00 |
| Quite a bit bothersome – Extremely bothersome | 1.13 | 0.29 | 3.89 | **0.001** |
| Moderately bothersome – Extremely bothersome | 1.04 | 0.30 | 3.42 | **0.007** |
| *Tinnitus annoyance change* |  |  |  |  |
| No tinnitus – Much less bothersome | -0.07 | 0.17 | -0.39 | 1.00 |
| No tinnitus – A little less bothersome | 0.64 | 0.16 | 4.10 | **<0.001** |
| No tinnitus – No change | 0.61 | 0.15 | 4.02 | **0.001** |
| No tinnitus – A little more bothersome | 1.08 | 0.25 | 4.35 | **<0.001** |
| No tinnitus – Much more bothersome | 1.55 | 0.25 | 6.24 | **<0.001** |
| No tinnitus – Don’t experience it before surgery | 1.45 | 0.36 | 4.02 | **0.001** |
| No tinnitus – Did not recall it before surgery | 1.32 | 0.27 | 4.92 | **<0.001** |
| Much less bothersome – A little less bothersome | 0.71 | 0.22 | 3.20 | **0.026** |
| Much less bothersome – No change | 0.68 | 0.22 | 3.11 | **0.035** |
| Much less bothersome – A little more bothersome | 1.15 | 0.29 | 3.91 | **0.002** |
| Much less bothersome – Much more bothersome | 1.62 | 0.29 | 5.50 | **<0.001** |
| Much less bothersome – Don’t experience it before surgery | 1.51 | 0.39 | 3.85 | **0.003** |
| Much less bothersome – Did not recall it before surgery | 1.38 | 0.31 | 4.46 | **<0.001** |
| A little less bothersome – No change | -0.03 | 0.21 | -0.13 | 1.00 |
| A little less bothersome – A little more bothersome | 0.44 | 0.28 | 1.55 | 0.752 |
| A little less bothersome – Much more bothersome | 0.91 | 0.28 | 3.19 | **0.027** |
| A little less bothersome – Don’t experience it before surgery | 0.80 | 0.39 | 2.08 | 0.390 |
| A little less bothersome – Did not recall it before surgery | 0.67 | 0.30 | 2.24 | 0.300 |
| No change – A little more bothersome | 0.47 | 0.28 | 1.65 | 0.683 |
| No change– Much more bothersome | 0.93 | 0.28 | 3.31 | **0.018** |
| No change – Don’t experience it before surgery | 0.83 | 0.38 | 2.16 | 0.341 |
| No change – Did not recall it before surgery | 0.70 | 0.30 | 2.34 | 0.243 |
| A little more bothersome – Much more bothersome | 0.47 | 0.34 | 1.36 | 0.854 |
| A little more bothersome – Don’t experience it before surgery | 0.36 | 0.43 | 0.84 | 0.988 |
| A little more bothersome – Did not recall it before surgery | 0.23 | 0.36 | 0.65 | 1.00 |
| Much more bothersome – Don’t experience it before surgery | -0.10 | 0.43 | -0.24 | 1.00 |
| Much more bothersome – Did not recall it before surgery | -0.23 | 0.36 | -0.66 | 1.00 |
| Don’t experience it before surgery – Did not recall it before surgery | -0.13 | 0.44 | -0.30 | 1.00 |

P-value is from a Tukey pairwise test. Bold indicates statistically significant p<0.05.

**Supplementary Table S2**. Number (%) of unilateral and bilateral CI subjects by age group.

|  | **18-34**  **(*n* = 222)** | **35-44**  **(*n* = 213)** | **45-54**  **(*n* = 410)** | **55-64**  **(*n* = 594)** | **65-74**  **(*n* = 546)** | **75-95**  **(*n* = 320)** | **Missing**  **(*n* = 2)** | **p-value** |
| --- | --- | --- | --- | --- | --- | --- | --- | --- |
| Unilateral | 104 (46.8%) | 130 (61.0%) | 259 (63.2%) | 422 (71.0%) | 422 (77.3%) | 273 (85.3%) | 2 (100%) | **<0.001** |
| Bilateral | 118 (53.1%) | 83 (39.0%) | 151 (36.8%) | 172 (29.0%) | 124 (22.7%) | 47 (14.7%) | 0 (0%) |  |

P-value is from a chi-squared test excluding the missing category. Bold indicates statistically significant p<0.05.
